# A Peroxiredoxin From the *Haemaphysalis longicornis* Tick Affects Langat Virus Replication in a Hamster Cell Line

**DOI:** 10.3389/fcimb.2020.00007

**Published:** 2020-01-28

**Authors:** Kodai Kusakisako, Haruki Morokuma, Melbourne Rio Talactac, Emmanuel Pacia Hernandez, Kentaro Yoshii, Tetsuya Tanaka

**Affiliations:** ^1^Laboratory of Parasitology, Department of Disease Control, Graduate School of Veterinary Medicine, Hokkaido University, Sapporo, Japan; ^2^Laboratory of Infectious Diseases, Joint Faculty of Veterinary Medicine, Kagoshima University, Kagoshima, Japan; ^3^Department of Clinical and Population Health, College of Veterinary Medicine and Biomedical Sciences, Cavite State University, Indang, Philippines; ^4^Department of Pathological and Preventive Veterinary Science, The United Graduate School of Veterinary Science, Yamaguchi University, Yamaguchi, Japan; ^5^Laboratory of Public Health, Faculty of Veterinary Medicine, Hokkaido University, Sapporo, Japan

**Keywords:** tick, peroxiredoxins, tick-derived molecule in the host, host–virus interaction, langat virus

## Abstract

Ticks are hematophagous arthropods, and their blood feeding on vertebrate hosts is essential for their development. The vertebrate blood contains high levels of free iron that can react with oxygen in ticks, resulting in the production of hydrogen peroxide (H_2_O_2_), one of the reactive oxygen species. Peroxiredoxins (Prxs), H_2_O_2_-scavenging enzymes, take on an important role in the ticks' oxidative stress coping mechanism. Ticks also transmit several disease-causing pathogens, including tick-borne encephalitis virus (TBEV), in animals and humans. Therefore, the control of ticks and tick-borne pathogens is a key issue that needs to be addressed. Infection with an arthropod-borne flavivirus is known to induce oxidative stress in insect cells. We hypothesize that vector-derived Prxs could have an effect on the infection and/or replication of flaviviruses in the hosts, since ticks Prxs are possibly transmitted from ticks to their hosts. In this study, we established stable strains of baby hamster kidney (BHK) cells expressing two types of H_2_O_2_-scavenging Prxs from the hard tick *Haemaphysalis longicornis* (BHK-HlPrx and BHK-HlPrx2 cells). Although the infection of TBEV surrogate Langat virus (LGTV) did not induce H_2_O_2_ production in normal BHK cells, the mortality rate and the virus titer of LGTV infected BHK-HlPrx cells increased. In addition, HlPrx proteins in BHK cells can facilitate LGTV replication in cells, while HlPrx2 proteins in BHK cells cannot. The results also demonstrated that this facilitation of LGTV replication by the 1-Cys Prx in the BHK cells is not by scavenging H_2_O_2_ but by an unknown mechanism. In order to understand this mechanism, more studies using tick-derived cells and ticks are necessary.

## Introduction

Ticks are obligate hematophagous ectoparasites and require blood feeding throughout their life cycle, except in the egg stage. The process from blood-feeding to blood-digestion lead to providing nutrition to ticks and activating tick life cycles including development, molting, and embryogenesis (Grandjean, 1983). Ticks feed on vertebrate blood that contains high levels of iron, like heme, and ferrous iron (Galay et al., 2014). In addition, ticks concentrate host-derived blood with iron, leading to a high concentration of iron in ticks. The concentrated iron can react with oxygen in the tick's body, resulting in high levels of reactive oxygen species (ROS), including hydrogen peroxide (H_2_O_2_) (Citelli et al., 2007; Galay et al., 2015). A high concentration of H_2_O_2_ causes oxidative damage to biopolymers, such as membrane lipids, nucleic acids, and proteins, leading to harmful effects on aerobic organisms (Robinson et al., 2010). Ticks have antioxidant enzymes that scavenge H_2_O_2_, like peroxiredoxins (Prxs) (Tsuji et al., 2001; Kusakisako et al., 2016a), catalases (Kumar et al., 2016), and selenoproteins (Adamson et al., 2014). Reports have demonstrated that the knockdown of these H_2_O_2_-scavenging enzyme genes has an inhibition effect on tick blood feeding and/or reproduction (Adamson et al., 2014; Kumar et al., 2016; Kusakisako et al., 2016a). Therefore, the antioxidant enzymes controlling the H_2_O_2_ concentration in ticks can be considered to be important in tick blood feeding and reproduction (Kusakisako et al., 2018a).

On the other hand, ticks are vectors of pathogens with economic importance to humans and animals, such as viruses, protozoa, and bacteria (Hoogstraal, 1985). H_2_O_2_-scavenging enzymes, such as Prxs, catalases, and selenoproteins, also have been analyzed in ticks to comprehend the interaction between the antioxidant enzymes and tick-borne pathogens (Narasimhan et al., 2007; Budachetri et al., 2017a,b). A Prx homologous protein (Salp25D) derived from the salivary glands of an *Ixodes scapularis* tick facilitates *Borrelia* to escape from neutrophil oxidation in the vertebrate host, resulting in successful transmission of the parasites from tick to host (Narasimhan et al., 2007). In addition, silencing of the *catalase* gene and inhibition of that protein resulted to the low transmission of *Rickettsia parkeri* to eggs of *Amblyomma maculatum* ticks (Budachetri et al., 2017b). Furthermore, the gene silencing of a selenocysteine insertion sequence (SECIS) binding protein (*SBP2*), involved in selenoprotein synthesis, significantly diminished the transovarial transmission of *R. parkeri* parasites to eggs in *A. maculatum* ticks (Budachetri et al., 2017a). Thus, these enzymes are also important to the vector competency of ticks with regard to horizontal transmission and transovarial transmission (Kusakisako et al., 2018a; Hernandez et al., 2019).

Tick-borne flaviviruses (TBFVs) induce considerable disease and death worldwide. Infections are characterized by mild to severe neurological symptoms, like meningitis and encephalitis (Weber et al., 2014; Mlera et al., 2015). In Europe, Russia, and Far East, including Japan, tick-borne encephalitis virus (TBEV) is considered one of the most medically important arboviruses, with 10,000 to 15,000 cases recorded each year (Lindquist and Vapalahti, 2008; Weber et al., 2014). Since most TBFVs require at least a biosafety level 3 (BSL3) containment facility, use of Langat virus (LGTV), a TBFV of low neurovirulence, provides a convenient BSL2 model of TBEV and other highly pathogenic TBFVs (Mlera et al., 2015). The Dengue virus, a known mosquito-borne flavivirus, requires the *catalase* gene to invade the mosquito midgut (Oliveira et al., 2017). Furthermore, some reports have demonstrated that mammalian cells which were infected with some arbovirus, such as Togaviridae, or expressing nonstructural proteins derived from TBEV were induced the production of ROS (Kuzmenko et al., 2016; Camini et al., 2017). These reports suggest that the H_2_O_2_-scavenging enzymes could also be important in the vector competency of arthropod-borne viruses.

Among these H_2_O_2_-scavenging enzymes in ticks, Prxs have been well-characterized in tick biology and in relationship to tick pathogens (Tsuji et al., 2001; Narasimhan et al., 2007; Kusakisako et al., 2016a, 2018a,b). Furthermore, Narasimhan et al. (2007) demonstrated that one tick Prx facilitate the transmission of pathogen from ticks to their host. Therefore, we considered that tick Prxs could be important for the interaction between ticks and tick-borne pathogens in the host. In this study, we established tick Prxs–expressing mammalian cells and investigated the interaction between tick-derived Prxs and LGTV infection in the mammalian cells.

## Materials and Methods

### Cell Culture and Virus

Baby hamster kidney (BHK-21) cells (ATCC CCL-10) were maintained in Eagle's minimum essential medium (EMEM) (Wako Pure Chemical Industries, Ltd., Osaka, Japan) containing 5% fetal bovine serum (FBS) (Equitech-Bio, Kerrville, TX, USA) and 1% antibiotic/antimycotic (Nacalai Tesque, Kyoto, Japan). The cells were maintained at 37°C under 5% CO_2_ until use.

The LGTV TP21 used in this study was amplified in BHK cells, and the virus stock titer was determined via focus forming assay as previously described (Talactac et al., 2016). The virus stock was aliquoted and stored at −80°C until use.

### Construction of *Haemaphysalis longicornis* Tick-Derived Prx Gene Expression Vectors Using the *H. longicornis actin* (*HlAct*) Promoter Region

Prxs can be classified into two groups in accordance with the presence of one or two conserved cysteines, 1-Cys or 2-Cys Prxs (Hall et al., 2011). In hard ticks, especially *H. longicornis*, some previous reports on tick Prxs have focused on 1-Cys Prx (HlPrx) (Tsuji et al., 2001), and 2-Cys Prx (HlPrx2) (Kusakisako et al., 2016a,b).

To construct *H. longicornis* tick-derived Prx (*HlPrx* and *HlPrx2*) gene expression vectors using an *HlAct* promoter region, the human phosphoglycerate kinase (PGK) promoter of the pmirGLO plasmid (Promega, Madison, WI, USA) was replaced with an *HlAct* promoter region as previously described (Kusakisako et al., 2018b), with some modifications. The reason we constructed the gene expression vectors using a tick-derived promoter was that tick-derived molecules were considered to be compatible with the tick-derived promoter for foreign protein expression in the host cells. The pmirGLO vector was double digested using *Bgl*II and *Xho*I, and the PGK promoter and luciferase sequence were removed from the pmirGLO plasmid (pmirGLO-no pro-no Luc). The plasmid was purified using the NucleoSpin® Gel PCR Clean-up Kit (Macherey-Nagel, Düren, Germany). The *HlAct* promoter region or *HlPrx* and *HlPrx2* genes with a FLAG-tag were amplified by polymerase chain reaction (PCR) using KOD-Plus-Neo (Toyobo, Osaka, Japan) with pmirGLO-*Bgl* II-*HlAct*-F and pmirGLO-*HlAct*-kozak-FLAG-R primers for the *HlAct* promoter region, FLAG-HlPrx-F and HlPrx-*Xho* I-R primers for the *HlPrx* gene, and FLAG-HlPrx2-F and HlPrx2-*Xho* I-R primers for the *HlPrx2* gene (Table 1). These PCR products were purified using the NucleoSpin® Gel PCR Clean-up Kit (Macherey-Nagel). The pmirGLO-no pro-no Luc plasmid and the purified PCR products were mixed with a 5× Infusion Enzyme (Takara, Shiga, Japan) and incubated at 50°C for 15 min. The pmirGLO-*HlAct* pro-*HlPrx* and pmirGLO-*HlAct* pro-*HlPrx2* plasmids were transformed into an *Escherichia coli* stellar strain, and the plasmid was then increased and purified using the Qiagen® Plasmid Midi Kit (Qiagen, Hilden, Germany) as previously described (Kusakisako et al., 2018b).

**Table 1:** Oligonucleotide primer sequences used for the construction of plasmids.

**Primer**	**Sequence (5^**′**^ → 3^**′**^)**
pmirGLO-*Bgl* II-*HlAct*-F	AGAGGATCGAGATCTGGCTTCGGACGAAGGCC
pmirGLO-*HlAct*-Kozak-FLAG-R	CTTGTCGTCGTCGTCCTTGTAGTCCAT$\underline{\underline{\text{GTTG}}}$ACTGT TTAGCTGCA
FLAG-HlPrx-F	ATGGACTACAAGGACGACGACGACAAGGGCGGCGGCATGCCTCCC
HlPrx-*Xho* I-R	GACTCTAGACTCGAGCTAATCCATGGTGGTGCGAAG GTAC
FLAG-HlPrx2-F	ATGGACTACAAGGACGACGACGACAAGGGCGGCGGCATGGACGTG
HlPrx2-*Xho* I-R	GACTCTAGACTCGAGCTATTGTTTGGCGAAGTAGGCC

*Underlines denote the restriction enzyme recognition site that was included in its primer name. A double underline denotes the estimated Kozak consensus sequence in Haemaphysalis longicornis. Broken underlines denote the spacer sequence*.

### Transfection of Plasmid Vectors Into BHK Cells and Establishment of HlPrx-Expressed BHK Cells Using Antibiotic G418

BHK cells were seeded in a 6-well plate at 2 ml/well of 1.5 × 10^5^ cells/ml and incubated at 37°C overnight. The plasmid vector (6 μg/well), 120 μl of Opti-MEM (Gibco, Grand Island, NY, USA), and 12 μl of HilyMax (Dojindo, Kumamoto, Japan) were mixed and incubated at room temperature (RT) for 15 min. Then the incubated mixture was added to the culture medium in each well, and the cells were incubated at 37°C for 16 h. After 16 h, 2 ml of the medium was added to each well, and the cells were further incubated for 32 h.

After transfection of the HlPrx-expressed plasmids to BHK cells, the supernatant was removed, and the medium with 1 mg/ml G-418 Sulfate Solution (Geneticin, Wako Pure Chemical Industries, Ltd.) was added every third day until the cells were confluent (around 10 days). G418 was used for the drug selection, since the pmirGLO plasmid originally has a G418-resistant gene. The confluent cells were transferred into a 96-well plate at 0.1 ml/well of 8 cells/ml and incubated at 37°C for 3 days. After 3 days, 0.1 ml of the medium with 0.3 mg/ml G418 was added to each well. The supernatant was replaced, and the medium containing 0.3 mg/ml G418 was replaced every third day until the single cell was colonized. The single-cell colony was transferred to a larger culture plate and flask. Finally, the drug-selected and HlPrx-expressing BHK cells (BHK-HlPrx and BHK-HlPrx2, respectively) were obtained. In addition, BHK cells used as a control cells following experiments did not go through the transfection process as described above.

### Protein Extraction and Western Blotting

To confirm the FLAG-tagged HlPrx and HlPrx2 proteins in BHK cells, immunostaining was performed. The obtained BHK cells were collected and suspended in phosphate buffered saline (PBS) and sonicated for 6 min at 45 kHz using a VS-100III ultrasonic cleaner (AS ONE Corporation, Osaka, Japan) and then centrifuged at 22,140 ×*g*. The supernatant was resolved in SDS-polyacrylamide gel electrophoresis (SDS-PAGE) gel under reducing conditions. After SDS-PAGE, the proteins were transferred onto a polyvinylidene difluoride (PVDF) membrane (Immobilon®-P, Millipore, Danvers, MA, USA). The membranes were blocked for 1 h at RT with 0.3% skim milk in PBS containing 0.05% Tween 20 (PBS-T, blocking solution); they were incubated with 1:1,000 dilutions of Anti-DDDDK-tag pAb (rabbit, MBL, Nagoya, Japan) against FLAG-tagged HlPrx; and HlPrx2 proteins in a blocking solution at 4°C overnight. For loading control, α-tubulin was detected using a monoclonal anti-α-tubulin antibody (mouse, Sigma-Aldrich, St. Louis, MO, USA). After washing three times in PBS-T, the membranes were incubated with a 1:50,000 dilution of horseradish peroxidase (HRP)-conjugated goat anti-rabbit or anti-mouse immunoglobulins (Dako, Glostrup, Denmark) in a blocking solution at RT for 1 h. After washing three times in PBS-T, bands were detected using Amersham^TM^ ECL^TM^ Prime Western Blotting Detection Reagent (GE Healthcare, Buckinghamshire, UK) and viewed using FluorChem® FC2 software (Alpha Innotech, San Leandro, CA, USA).

### Detection of H_2_O_2_ Using BES-H_2_O_2_-Ac in BHK Cells

The intracellular H_2_O_2_ detection in BHK cells was performed using BES-H_2_O_2_-Ac (Wako Pure Chemical Industries, Ltd.) as previously described (Kusakisako et al., 2018b), with some modifications. BHK, BHK-HlPrx, and BHK-HlPrx2 cells were seeded in a 24-well plate at 500 μl/well of 1.5 × 10^5^ cells/ml and incubated overnight at 37°C. After removing the supernatants, the cells were washed with 500 μl of PBS in each well. After washing the cells, the supernatants were replaced with 5 μM BES-H_2_O_2_-Ac and 1 μM Hoechst 33342 (Dojindo) in a culture medium without FBS and incubated at 37°C for 30 min. The cells were washed again with PBS, and then the supernatants were replaced with 0.05% H_2_O_2_ in a culture medium and incubated at 37°C for 30 min. After exposure to H_2_O_2_, the cells were washed with PBS, and the supernatants were replaced with the culture medium. The cells were observed under a fluorescent microscope (IX71, Olympus, Tokyo, Japan). Furthermore, to measure the fluorescence intensities of H_2_O_2_ in the cells, the cells were collected using 120 μl of 0.25% Trypsin-EDTA solution (Wako Pure Chemical Industries, Ltd.). The collected cells (100 μl) were transferred to a 96-well plate to measure their fluorescence. Fluorescence was detected using a microplate reader (SH-9000Lab, Corona Electric, Ibaraki, Japan) with excitation at 480 nm and emission at 535 nm for BES-H_2_O_2_-Ac and with excitation at 352 nm and emission at 461 nm for Hoechst 33342.

### Cell Survival Assays After the H_2_O_2_ Treatment of BHK Cells

After H_2_O_2_ treatment, the BHK cells (20 μl) were used for *in vitro* cell survival assay (Strober, 2001). The cells were mixed with 20 μl of Trypan blue (Nacalai Tesque). Then the ratio between the surviving and dead Trypan blue–stained cells was determined using a hemocytometer.

### Cell Survival Assays After LGTV Infected BHK Cells

To measure the cell survival rate of the LGTV-infected BHK cells and the BHK-HlPrx and BHK-HlPrx2 cells, the normal BHK, BHK-HlPrx, and BHK-HlPrx2 cells were seeded in a 24-well plate at 500 μl/well of 4.0 × 10^5^ cells/ml and incubated overnight at 37°C. The supernatants were replaced with a culture medium containing 0.01 multiplicity of infection (MOI) of LGTV and incubated for 1 h at 37°C. For each assay, cells were either infected with LGTV or were mock-infected with control medium. The cells were washed with PBS to remove the unabsorbed viruses. Then the plates were incubated at 37°C for 3 days. To collect the dead cells during this experiment, the supernatants were collected and centrifuged at 100 ×*g*. The supernatants for the measurement of virus titers were collected in new tubes and stored at −30°C until use. The live cells attached to the plate were collected using 100 μl of 0.25% Trypsin-EDTA (Wako Pure Chemical Industries, Ltd.). Finally, the centrifuged detached cells and the collected attached cells were mixed. The counting of the live and dead cells was performed as mentioned above.

### Titration of LGTV in the Supernatant Using a Focus Forming Assay

The LGTV used in this study was amplified in BHK, BHK-HlPrx, and BHK-HlPrx2 cells, and the virus titers were determined by focus forming assay as previously described (Talactac et al., 2016). Briefly, serial 10-fold dilutions of the cultured supernatants were plated on 4.0 × 10^5^ cells/well of BHK cells in 24-well plates and incubated at 37°C for 1 h. After washing with PBS, the infected cells were overlaid with 1.5% methylcellulose containing modified Eagle's medium (MEM) (Gibco) with 1% FBS and 1% antibiotic/antimycotic and incubated at 37°C for 4 days. The supernatants were removed, and the cells were fixed by a 4% paraformaldehyde phosphate buffer solution (pH 7.4) at RT for 30 min. The fixed cells were blocked by 5% skim milk in PBS at RT for 1 h. After washing with PBS, viral foci were detected by a primary antibody against Langat virus in mice (1:1,000 dilution), followed by Alexa Fluor® 594 goat antimouse IgG (1:1,000 dilution, Invitrogen, Carlsbad, CA, USA). For taking photos, we decided the maximum exposure time using the LGTV uninfected BHK cells, and then, the photos of the experimental groups were taken under the maximum exposure time. The foci were counted using a fluorescence microscope (IX71, Olympus), and the virus titers of the cultured supernatant derived from BHK, BHK-HlPrx, and BHK-HlPrx2 cells were expressed as focus-forming unit (FFU) per milliliter (FFU/ml).

### Statistical Analysis

All experiments were done at least three times. The results are shown as average ± standard deviation (SD). A one-way ANOVA test was applied to the obtained data, and statistically significant differences (*p* < 0.001) in each group were demonstrated. For pair comparisons within groups, Tukey's test was applied. In contrast, the statistical comparison between two groups of the same cells was analyzed using Welch's *t*-test. *P* < 0.05 was considered to be a statistically significant difference.

## Results

### Detection of FLAG-Tagged HlPrxs in Transfected BHK Cells Using Western Blotting

**Figure 1:**
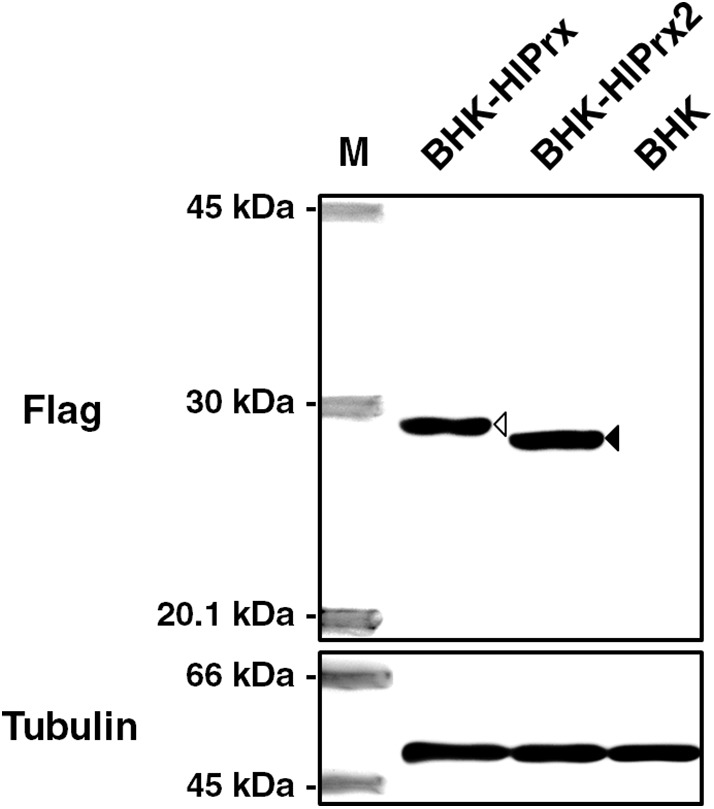
Detection of FLAG-tagged HlPrxs in transfected BHK cells using Western blotting. The left column indicates the specific antibody against FLAG-tagged HlPrx and HlPrx2 proteins or α-tubulin in BHK cells. The upper rows show the BHK cell lines. The white arrowhead indicates the FLAG-tagged HlPrx protein (~25.7 kDa), while the black arrowhead indicates the FLAG-tagged HlPrx2 protein (~23.5 kDa). The α-tubulin protein served as a loading control. M, molecular weight Marker; BHK-HlPrx, *Haemaphysalis longicornis* 1-Cys peroxiredoxin-expressing baby hamster kidney cells; BHK-HlPrx2, *H. longicornis* 2-Cys peroxiredoxin-expressing baby hamster kidney cells.

To detect FLAG-tagged HlPrx and HlPrx2 in the transfected BHK cells (BHK-HlPrx and BHK-HlPrx2 cells), Western blotting was performed using an anti-FLAG-tag antibody as a primary antibody. Western blotting demonstrated that the FLAG-tagged HlPrx and HlPrx2 in BHK cells were detected with molecular weights of 25.7 kDa (white arrowhead) and 23.5 kDa (black arrowhead), respectively (Figure 1). On the other hand, in the normal BHK cells, FLAG-tagged HlPrxs were not detected. These results demonstrated that the HlPrx- or HlPrx2-expressing plasmid-transfected BHK cell lines (BHK-HlPrx and BHK-HlPrx2 cells) expressed a certain Prx protein in the cells.

### Antioxidant Activity of BHK-HlPrx and BHK-HlPrx2 Cells Against H_2_O_2_ Exposure

**Figure 2:**
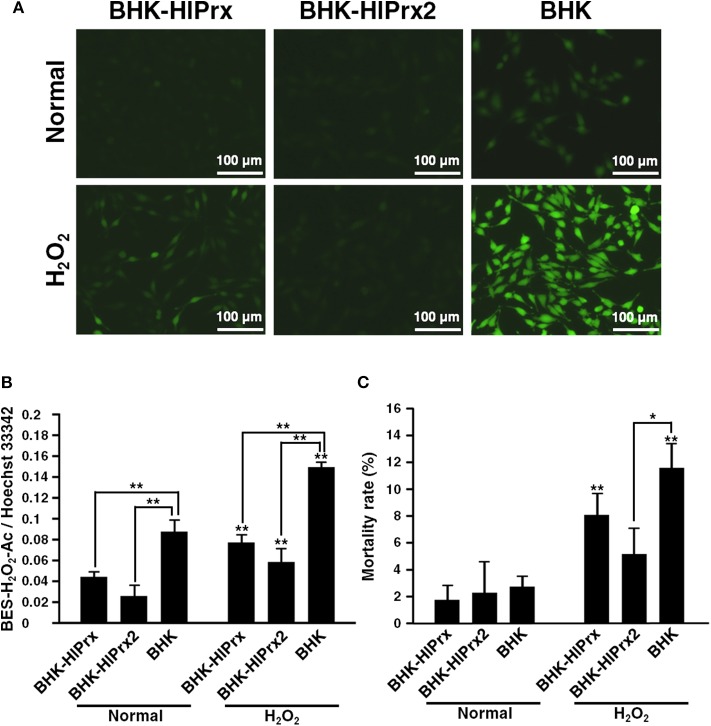
Antioxidant activity of BHK-HlPrx and BHK-HlPrx2 cells against H_2_O_2_ exposure. **(A)** The fluorescence of the BES-H_2_O_2_-Ac probe in BHK-HlPrx, BHK-HlPrx2, and BHK cells was observed under a fluorescence microscope after H_2_O_2_ treatment. The left column indicates the treatment of the cells. Scale bars: 100 μm. **(B)** Graph of the fluorescence intensities of the BES-H_2_O_2_-Ac probe in the BHK cell lines after exposure to 0.05% H_2_O_2_ for 30 min. The intensities are shown as the ratio of BES-H_2_O_2_-Ac/Hoechst 33342 intensities. **(C)** Graph of the mortality rate in BHK cell lines after exposure to 0.05% H_2_O_2_ for 30 min. In the **(B,C)** graph, the results are shown as average ± standard deviation (SD). Data were analyzed using Tukey's test in the same state as indicated by the line with asterisks and Welch's *t*-test in the same BHK cell line as indicated by the line with asterisks above error bars in the H_2_O_2_-treated state. *P < 0.05 and **P < 0.01 indicate significant differences. Normal, normal cultured state; H_2_O_2_, 0.05% H_2_O_2_-treated state; BHK-HlPrx, *H. longicornis* 1-Cys peroxiredoxin-expressing baby hamster kidney cells; BHK-HlPrx2, *H. longicornis* 2-Cys peroxiredoxin-expressing baby hamster kidney cells.

To evaluate the antioxidant activity of BHK-HlPrx and BHK-HlPrx2 cells against H_2_O_2_, the detection of intracellular H_2_O_2_ in the BHK cells was conducted using a BES-H_2_O_2_-Ac probe. The BHK cells were incubated with 5 μM BES-H_2_O_2_-Ac and 1 μM Hoechst 33342, and then the cells were exposed to 0.05% H_2_O_2_. The cells were observed under fluorescence microscopy, while the fluorescent intensities in BHK cells were measured using the microplate reader. Fluorescent microscopy revealed that the fluorescence of the intracellular H_2_O_2_ in the BHK-HlPrx and BHK-HlPrx2 cells was of lower intensity than that in BHK cells (Figure 2A, H_2_O_2_), even though without the addition of external H_2_O_2_, the H_2_O_2_ fluorescence in the BHK-HlPrx and BHK-HlPrx2 cells seemed to be weaker than that in the BHK cells (Figure 2B, Normal). In addition, the microplate reader fluorescence intensity measurement showed that the intracellular H_2_O_2_ in the BHK-HlPrx and BHK-HlPrx2 cells had significantly lower relative intensities as compared with the BHK cells in both the normal and H_2_O_2_-exposed states (Figure 2B). In each cell line, the fluorescence intensities of H_2_O_2_ in the BHK cells significantly increased with exposure to 0.05% H_2_O_2_ (Figure 2B, Normal vs. H_2_O_2_).

To evaluate the mortality rate among three cell lines cultured with or without H_2_O_2_, Trypan blue assays were conducted. The Trypan blue assays revealed that the mortality rate of BHK-HlPrx2 cells was significantly lower than that of BHK cells under the H_2_O_2_ treatment (Figure 2C, H_2_O_2_). In addition, the mortality rate of BHK-HlPrx and BHK cells in the H_2_O_2_-exposed state was significantly higher than in the normal state (Figure 2C). These results suggested that HlPrxs decrease the H_2_O_2_ concentration in the host cells, and HlPrx2 might have a higher antioxidant activity than HlPrx, resulting in a decreased mortality rate in BHK-HlPrx2 cells.

### Interaction Between Tick Prxs and LGTV in BHK Cells

**Figure 3:**
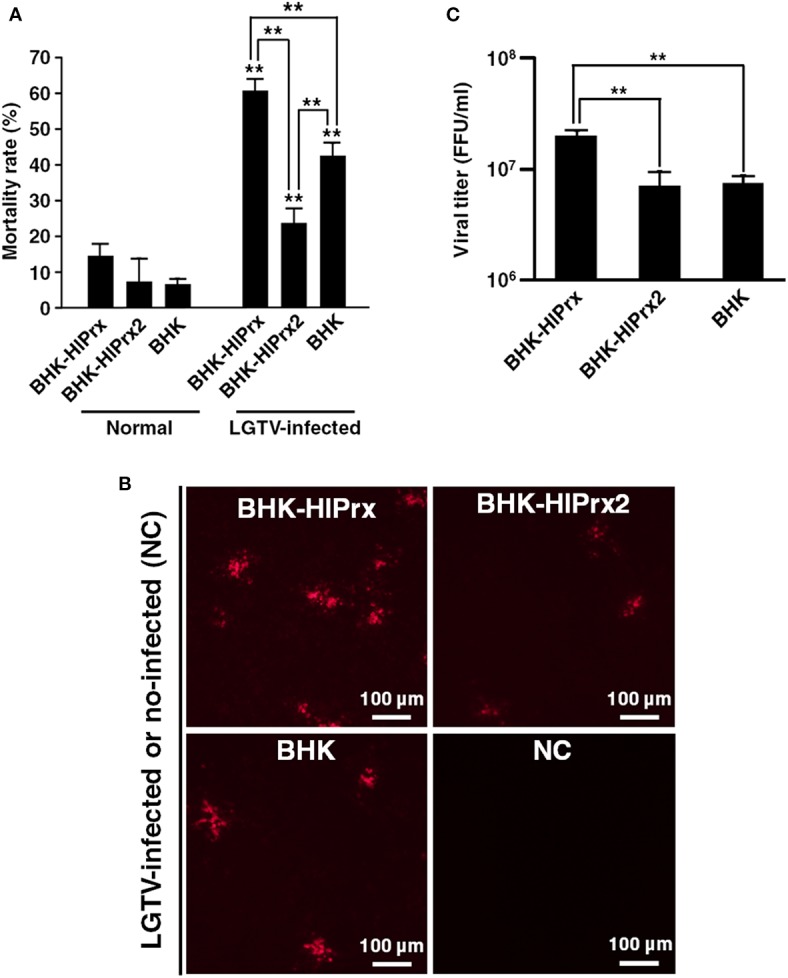
Interaction between tick Prxs and LGTV in BHK cells. **(A)** Graph of the mortality rate in BHK cell lines after infection with LGTV. **(B)** Observation of LGTV in BHK cell lines under fluorescent microscopy using anti-LGTV mouse antisera. Scale bars: 100 μm. NC, Negative Control for a baseline fluorescence. **(C)** Graph of the viral titers in cultured supernatants from the BHK cell lines infected with LGTV. Viral titers represented as foci-forming-unit (FFU)/ml. In the **(A,C)** graph, the results are shown as average ± standard deviation (SD). Data were analyzed using Tukey's test in the same state as described line with asterisks and Welch's *t*-test in the same BHK cell line as described asterisks above error bars in H_2_O_2_ treated state. **P < 0.01 indicate significant differences. Normal, normal cultured state; LGTV-infected, the cell lines infected with Langat virus; BHK-HlPrx, *H. longicornis* 1-Cys peroxiredoxin-expressing baby hamster kidney cells; BHK-HlPrx2, *H. longicornis* 2-Cys peroxiredoxin-expressing baby hamster kidney cells.

To evaluate the effects of LGTV infection on the mortality rate of BHK-HlPrx and BHK-HlPrx2 cells, Trypan blue assays were conducted after the BHK cell lines were infected with LGTV. Before conducting Trypan blue assays in these BHK cell lines, we confirmed whether the concentration of H_2_O_2_ will increase in BHK cells due to LGTV infection. First, we observed BHK cells infected with LGTV and treated with BES-H_2_O_2_-Ac and Hoechst 33342 under fluorescence microscopy. The fluorescence microscopy indicated that LGTV infection did not induce H_2_O_2_ production in LGTV-infected or the normal-state BHK cells (Supplementary Figure 1A). In addition, we evaluated the H_2_O_2_ fluorescence intensities in BHK cells using a microplate reader. We did not observe any difference in H_2_O_2_-specific fluorescent intensity between the normal state and LGTV-infected BHK cells (Supplementary Figure 1B). These results indicated that LGTV infection of BHK cells might not influence the concentration of H_2_O_2_. Therefore, we considered that the LGTV-infection also may not influence H_2_O_2_ concentrations in BHK-HlPrx and BHK-HlPrx2 cells. Next we conducted Trypan blue assays to evaluate the effects of LGTV infection on the mortality rate of BHK cell lines. The assays revealed that LGTV infection of the BHK cell lines significantly increased the mortality rate in all cell lines (Figure 3A). Although the mortality rate in BHK-HlPrx cells was significantly increased (142.7%) as compared with BHK cells, the mortality rate in BHK-HlPrx2 cells was significantly decreased (55.6%) as compared with BHK cells (Figure 3A, LGTV-infected; Supplementary Table 1).

Moreover, to compare the LGTV replication among BHK-HlPrx, BHK-HlPrx2, and BHK cells, the virus titers of the culture supernatants were measured. First, the LGTV-derived proteins were observed under a fluorescent microscope using anti-LGTV mouse antiserum. The fluorescence microscopy revealed that LGTV-derived fluorescence was detected in all culture supernatants, and more fluorescent foci were observed in the BHK-HlPrx compared to BHK-HlPrx2 or normal BHK cells (Figure 3B). In addition, when the virus titers were measured in the culture supernatants, a significantly higher virus titer was detected in the supernatant of BHK-HlPrx cells than in the supernatants of BHK-HlPrx2 and BHK cells (Figure 3C; Supplementary Table 1). However, the virus titer in the supernatant of BHK-H1Prx2 cells was not significantly different from the virus titer in the supernatant of BHK cells, although the cell mortality rate in BHK-HlPrx2 cells was significantly lower than the one in BHK cells (Figure 3). These results indicated that the HlPrx protein in BHK cells increases LGTV replication, leading to a higher mortality rate in BHK-HlPrx cells.

## Discussion

H_2_O_2_-scavenging enzymes in ticks, like Prxs, catalases, and selenoproteins, have been investigated to comprehend not only their antioxidant effects in ticks (Adamson et al., 2014; Kumar et al., 2016; Kusakisako et al., 2016a), but also their interactions with tick-borne pathogens (Narasimhan et al., 2007; Budachetri et al., 2017a,b). In *I. scapularis* ticks, 1-Cys Prx (Salp25D) is important for *B. burgdorferi* transmission from ticks to the host to protect *Borrelia* from neutrophil oxidation in the host (Narasimhan et al., 2007). In *I. ricinus* ticks, two Prx-homologous genes were strongly induced in the hemolymph during *B. burgdorferi* infection (Rudenko et al., 2005). In addition, the obstruction of the catalase functions or knockdown of the *SBP2* gene related to selenoprotein synthesis in *A. maculatum* resulted in the low transmission of *R. parkeri* to tick eggs (Budachetri et al., 2017a,b). Thus, these enzymes affect the transmission of tick-borne pathogens from ticks to hosts or from adult female ticks to their eggs.

In the present study, we utilized *H. longicornis* tick-derived Prxs-expressing BHK cell lines. The BHK cells themselves have four BHK cells-derived Prxs (Supplementary Table 2). Therefore, both the *H. longicornis* tick-derived Prx and BHK-cells derived Prxs are present within the cells, but on this study, we focused on the Prxs derived from *H. longicornis* ticks. Prxs can be divided into two groups (1-Cys and 2-Cys Prxs) based on the presence of one or two conserved cysteines (Hall et al., 2011). In general, 2-Cys Prxs have two conserved cysteines, peroxidatic and resolving ones (Hofmann et al., 2002). The peroxidatic cysteine reacts with and detoxifies H_2_O_2_. Then the post-reaction peroxidatic cysteine reacts with another Prx's resolving cysteine, and the two Prxs form homodimers via intermolecular disulfide bonds. These disulfide bonds are resolved by thioredoxins, and the reduced 2-Cys Prxs become active forms (Lu and Holmgren, 2014). On the other hand, 1-Cys Prxs contain only the peroxidatic cysteine without a resolving cysteine (Choi et al., 1998). The mechanism of 1-Cys Prxs is considered to react with and detoxify H_2_O_2_ as one molecule. Then the post-reaction peroxidatic cysteine is reduced by a hydrogen donor such as glutathione, and finally the enzyme is recycled (Wood et al., 2003). In addition, mammal Prxs are classified into six subgroups (Prx I to Prx VI) in accordance with the localization within the cells: Prx I and Prx II are localized in the cytosol, Prx III in mitochondria, Prx IV in the extracellular space, Prx V in mitochondria and peroxisomes, and Prx VI in the cytosol (Rhee et al., 2005). Prx I to Prx V are 2-Cys Prxs, and Prx VI is a 1-Cys Prx (Rhee et al., 2005). The tick Prxs used in this study were 1-Cys Prx (HlPrx) and 2-Cys Prx (HlPrx2). HlPrx was classified as Prx VI, and the identity with BHK cell-derived Prx VI, a 1-Cys Prx, was 62.8%, with 97% coverage against the Prx VI of BHK cells (Supplementary Table 2). HlPrx2 could be classified as Prx II, since the localization within tick hemocytes, a cell in tick hemolymph, is the cytosol (Kusakisako et al., 2016b) and the full length of the amino acid sequence is almost the same between HlPrx2 and Prx II (Supplementary Table 2). The identity with BHK cell-derived Prx II was 75.3%, with 96% coverage against the Prx II of BHK cells (Supplementary Table 1). In addition, the identity between HlPrx and HlPrx2 was 30.4%, with 75% coverage (Supplementary Table 2). These results suggested that HlPrx and HlPrx2 proteins expressed in the BHK-HlPrx or BHK-HlPrx2 cell lines would localize to the cytosol of the cells, and the functions against the cells might be different.

In this study, we were interested in the associated effects on the pathogen within the host cell in the presence of Prxs. We initially confirmed the biological activity of the expressed HlPrx and HlPrx2 proteins in the established BHK cell lines through the antioxidant activity against H_2_O_2_. The results (Figures 1, 2) demonstrated that the HlPrx and HlPrx2 proteins expressed in the established cell lines (BHK-HlPrx and BHK-HlPrx2 cells) had antioxidant activity through scavenging H_2_O_2_. The survival rate of the BHK-HlPrx2 cell line was higher than that of the other cell lines (Figure 2C). The HlPrx2 protein was reported to play an important role in the antioxidant activity in tick blood feeding and oviposition by controlling the H_2_O_2_ concentration in ticks (Kusakisako et al., 2016b). In addition, tick Prxs would transfer to their host body during tick blood feeding (Tsuji et al., 2001; Narasimhan et al., 2007; Tirloni et al., 2015; Kim et al., 2016). Therefore, the present study, together with other notable reports, indicates that the established cell lines (BHK-HlPrx and BHK-HlPrx2 cells) could be a model environment in which the tick-derived proteins interacted with tick-transmitted pathogens in the host cell.

To evaluate the interaction between BHK cells and LGTV on H_2_O_2_ induction in the host cells, we observed the fluorescence and amount of H_2_O_2_ in BHK cells infected with LGTV. However, there was no significant difference in the fluorescence and amount of H_2_O_2_ between the normal state and LGTV-infected BHK cells (Supplementary Figure 1). This suggested that LGTV infection does not induce H_2_O_2_ in BHK cells. On the other hand, it has been reported that a human liver-derived cell line infected with Togavirus, an arbovirus, were induced the production of ROS (Camini et al., 2017). Furthermore, Kuzmenko et al. (2016) reported that ROS induction was observed when non-structural proteins of TBEV were expressed in a human kidney-derived cell line. In other viruses, such as influenza A and lymphocytic choriomeningitis, infection of the host induced ROS production in granulocytes, including neutrophils (Akaike et al., 1996; Lang et al., 2013). These reports showed that the ROS production was induced by virus infections in cell lines, including the immune cells, such as neutrophils. In the present study, ROS induction was not observed in BHK cells infected with LGTV. It could be that LGTV did not stimulate ROS production in BHK cells or that the stimulation of ROS production in BHK cells by LGTV occurred minimally. Therefore, it is expected that further evaluation of the interaction between the viral infection and HlPrxs protein is possible by using the immune cells, such as neutrophils and macrophages, in which the induction of ROS production by viral infection has been reported.

Interestingly, our experiments demonstrated that the mortality rate of the LGTV-infected BHK-HlPrx cells and the virus titer of the culture supernatant from those cells increased, even though LGTV infection did not induce the production of H_2_O_2_ in the virus-infected BHK-HlPrx cells (Figure 3; Supplementary Figure 1). These results suggested that an unknown mechanism in HlPrx promotes the growth of LGTV in BHK-HlPrx cells. In general, flaviviruses replicate on the endoplasmic reticulum (ER), including the lysosome, after invading the target cells (Okamoto et al., 2017). 1-Cys Prx in mammalian cells is known to be localized in the cytoplasm and lysosome (Fisher, 2017), and thus, 1-Cys Prx would be abundant in the ER that produces lysosome. In addition, cyclophilin A, one of the immunophilins, facilitates the replication of flaviviruses to interact with virus-derived non-structural proteins (Qing et al., 2009), and cyclophilin A would be related to 1-Cys Prx (Prx VI) (Ishii et al., 2012). These reports suggested the possibility that 1-Cys Prx affects the replication of flaviviruses on the ER. On the other hand, LGTV infection is known to be involved in the apoptosis control system through the upregulation of caspase-3 and -7 in human embryonic kidney (HEK) 293T cells (Mlera et al., 2016). Dengue fever virus (DENV) infection was also reported to induce mitochondria-mediated apoptosis in BHK cells and Vero cells derived from a monkey kidney (Nasirudeen et al., 2008). In addition, the ER stress due to infection with the Japanese encephalitis virus (JEV) promoted apoptosis in BHK cells (Huang et al., 2016). Flavivirus-related apoptosis is often induced by virus-derived proteins, such as capsid proteins and non-structural proteins (Bhuvanakantham et al., 2010). These reports and our results suggest that the promotion of LGTV replication in BHK-HlPrx cells led to the induction of apoptosis of the infected cells and a higher mortality rate among the experimental cell lines.

In BHK-HlPrx2 cells, the mortality due to LGTV infection significantly decreased as compared to normal BHK cells, while the virus titer from the culture supernatant did not increase (Figure 3). The HlPrx2 protein in ticks is considered to be important for tick survival and development through the control of H_2_O_2_ concentration during and after blood feeding (Kusakisako et al., 2016b). However, the H_2_O_2_ concentration in BHK cells, including the BHK-HlPrx and BHK-HlPrx2 cell lines, would not increase (Supplementary Figure 1). In ticks, the HlPrx2 protein localizes inside the cell and tissue membranes using immunostaining (Kusakisako et al., 2016b). In addition, HlPrx2 proteins can become a hexamer with a chaperon activity (Moon et al., 2005; Hall et al., 2011; König et al., 2013; Kusakisako et al., 2016b), which would lead to protection of the membrane from damage, including oxidative stress and viral infection. Therefore, we concluded that the HlPrx2 protein in the BHK-HlPrx2 cell line acted to maintain homeostasis in the BHK cells, resulting in lower mortality of the BHK-HlPrx2 cells infected with LGTV in comparison with the BHK cells infected with LGTV. These results and reports also suggested that HlPrx2 proteins have a low possibility of being related to LGTV replication in the BHK cells.

Chen et al. (2018) reported that DENV replicates and induces ROS production in a mosquito-derived cell line without a cytopathic effect (CPE), while the infection of a mammalian cell line leads to cell death. Likewise, although LGTV infection of BHK cells induced CPE in the cells at Day 3 after inoculation, LGTV could replicate in an *I. scapularis* tick cell line (ISE6) without inducing CPE in the cells (Mlera et al., 2016). Additionally, many immune-associated pathways in *I. scapularis*/*I. ricinus* cells are observed with protein expression changes following TBFV infection (Weisheit et al., 2015; Grabowski et al., 2016). These reports and the results from this study indicate that LGTV infection of mammalian cells and tick cells would produce different reactions with different immune responses; thus, we should consider similar experiments using ISE6 cells infected with LGTV to evaluate the interaction between tick-derived Prxs and LGTV in tick cells. It would also be of interest and could be a subject of future studies if the same result could be observed if a more virulent TBFV such as TBEV is infected on both mammalian and tick cells.

As the purpose of this study was evaluation of the interaction between LGTV infection and HlPrxs, which are tick-derived molecules, we established the HlPrxs expressing BHK cell lines and investigated the effects of LGTV infection of these cell lines. The virus replication of LGTV and the cell mortality of BHK cell lines infected with LGTV were evaluated using the tick-derived molecules expressing BHK cells (BHK-HlPrx and BHK-HlPrx2 cells). These results suggest that LGTV might utilize the tick Prxs to facilitate replication in the host cells. This study is considered to be an important model to elucidate the interaction between tick-derived molecules and tick-borne pathogens in the host.

## Data Availability Statement

All datasets generated for this study are included in the article/Supplementary Material.

## Author Contributions

KK, HM, and TT designed the study and interpreted the data. KK and HM collected the data and wrote the manuscript. KK, HM, MT, EH, KY, and TT analyzed the data. MT, EH, KY, and TT revised and approved the manuscript.

### Conflict of Interest

The authors declare that the research was conducted in the absence of any commercial or financial relationships that could be construed as a potential conflict of interest.

## Supplementary Material

Supplementary Figure 1 Evaluation of the effects of LGTV infection on H_2_O_2_ concentration in BHK cells. **(A)** Observation of H_2_O_2_ in BHK cells infected with LGTV under fluorescent microscopy using BES-H_2_O_2_-Ac. Scale bars: 100 μm. **(B)** Graph of the fluorescence intensities of the BES-H_2_O_2_-Ac probe in BHK cells infected with LGTV. The fluorescent intensities are shown as the ratio of BES-H_2_O_2_-Ac/Hoechst 33342 intensities. Data were analyzed using Tukey's test. ^**^*P* < 0.01 indicates significant differences. Normal, normal cultured state as a negative control; LGTV-infected, BHK cells infected with Langat virus; H_2_O_2_, 0.05% H_2_O_2_-treated state as a positive control.

Supplementary Table 1 Summary of the results on mortality rate and viral titer in BHK cell lines infected with LGTV.

Supplementary Table 2 Comparison of Prxs derived from H. Longicornis ticks and BHK cells on amino acid sequences.
