# Supplementary figures and images for: A Peroxiredoxin From the Haemaphysalis longicornis Tick Affects Langat Virus Replication in a Hamster Cell Line
Source: Front Cell Infect Microbiol. 2020 Jan 28;10:7. doi: 10.3389/fcimb.2020.00007 (PMC6997474; doi:10.3389/fcimb.2020.00007)

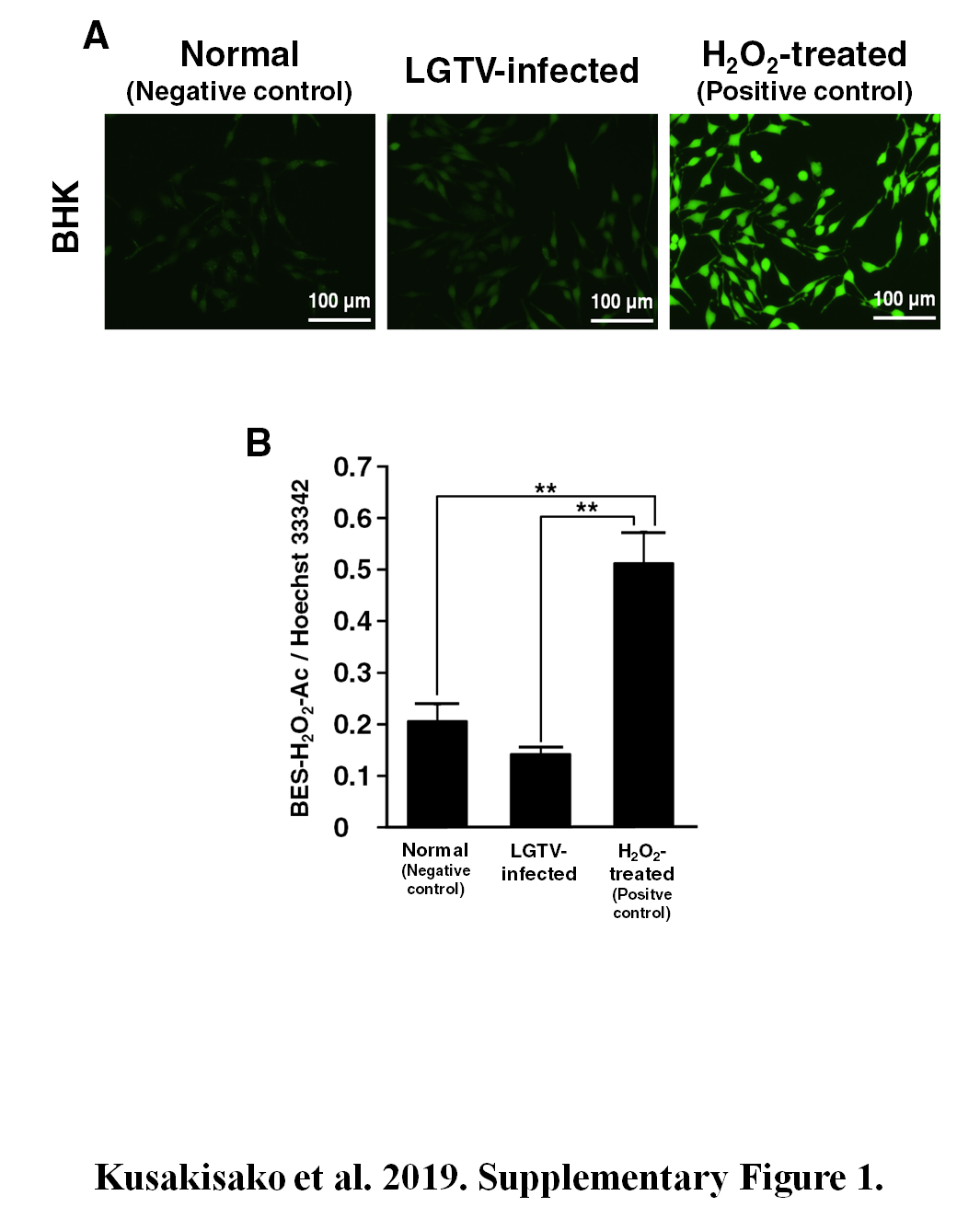

Supplement: Supplementary Figure 1 — Evaluation of the effects of LGTV infection on H2O2 concentration in BHK cells. (A) Observation of H2O2 in BHK cells infected with LGTV under fluorescent microscopy using BES-H2O2-Ac. Scale bars: 100 μm. (B) Graph of the fluorescence intensities of the BES-H2O2-Ac probe in BHK cells infected with LGTV. The fluorescent intensities are shown as the ratio of BES-H2O2-Ac/Hoechst 33342 intensities. Data were analyzed using Tukey's test. **P < 0.01 indicates significant differences. Normal, normal cultured state as a negative control; LGTV-infected, BHK cells infected with Langat virus; H2O2, 0.05% H2O2-treated state as a positive control. [file Image_1.TIF]
