# Supplementary material for: A Peroxiredoxin From the Haemaphysalis longicornis Tick Affects Langat Virus Replication in a Hamster Cell Line
Source: Front Cell Infect Microbiol. 2020 Jan 28;10:7. doi: 10.3389/fcimb.2020.00007 (PMC6997474; doi:10.3389/fcimb.2020.00007)
Supplement: Supplementary Table 1 — Summary of the results on mortality rate and viral titer in BHK cell lines infected with LGTV. [file Data_Sheet_1.docx]

Supplementary Material

## Supplementary Table

**Supplementary Table 1 Summary of the results on mortality rate and viral titer in BHK cell lines infected with LGTV**

| Cell lines |  | Mortality rate | |  | Viral titer | |
| --- | --- | --- | --- | --- | --- | --- |
|  |  | Obtained data (%) | Fold change vs BHK |  | Obtained data (FFU/ml) | Fold change vs BHK |
| BHK-HlPrx |  | 60.7 ± 3.3** | 142.7% |  | 20,190,000 ± 2,217,589** | 263.5% |
| BHK-HlPrx2 |  | 23.6 ± 4.1** | 55.6% |  | 7,306,667 ± 2,002,407** | 95.3% |
| BHK |  | 42.5 ± 3.6 | - |  | 7,663,333 ± 1,083,990 | - |

The obtained data were shown as mean ± standard division (SD). The fold change vs BHK indicate how increased or decreased as compared with the BHK cell line, respectively. ***P* < 0.01 indicates significant difference vs BHK cell line. BHK-HlPrx, *Haemaphysalis longicornis* 1-Cys peroxiredoxin-expressing baby hamster kidney cells; BHK-HlPrx2, *H. longicornis* 2-Cys peroxiredoxin-expressing baby hamster kidney cells.

**Supplementary Table 2 Comparison of Prxs derived from *H. longicornis* ticks and BHK cells on amino acid sequences**

| Identities using blastp | BHK cells (*Mesocricetus auratus*) | | | | HlPrx |
| --- | --- | --- | --- | --- | --- |
|  | Prx I (235 aa)  [XP_021084022] | Prx II (198 aa)  [XP_005087380] | Prx IV (274 aa)  [XP_005078033] | Prx VI (224 aa)  [XP_005071394] |  |
| HlPrx  (222 aa)  [BAB17604] | 29.9%  (80%) | 31.3%  (80%) | 28.4%  (69%) | 62.8%  (97%) | ― |
| HlPrx2  (197 aa)  [BAU79645] | 74.0%  (96%) | 75.3%  (96%) | 75.3%  (96%) | 30.7%  (89%) | 30.4%  (75%) |

The numbers with parentheses and “aa” after the Prx names indicate the full length of the amino acid sequences. The numbers with square brackets indicate accession numbers of NCBI Reference Sequence or GenBank. The percentages without or with parentheses indicate the percentage of identity or coverage against query sequences on blastp, respectively. 1-Cys Prx = Prx VI and HlPrx; 2-Cys Prx = Prx I, Prx II, Prx IV, and HlPrx2.
